# Research on the Influence Mechanism of Personal Initiative on Enterprise Emergency Management Ability

**DOI:** 10.3389/fpsyg.2021.618034

**Published:** 2021-11-10

**Authors:** Shuang Li, Feng Xu, Zhengquan Xu, Yuqing Kang, Qifeng Yang

**Affiliations:** School of Economics and Management, China University of Mining and Technology, Xuzhou, China

**Keywords:** executive ability, perceived organizational support, political skills, emergency management ability, personal initiative

## Abstract

This study aims to explore the influence mechanism of personal initiative on the overall emergency management ability of enterprises so as to put forward effective measures to improve the emergency management ability. Based on social interaction theory and feature activation theory, the concepts of organizational support theory, executive power, and political skills were introduced to construct a corresponding theoretical model. We collected data through an online questionnaire to test this model *via* structural equation model analysis and regression analysis, with 208 participants of varying backgrounds. The results show that personal initiative can strengthen enterprise emergency management ability. The mediating effect of executive power between personal initiative and emergency management ability of enterprise has also been proved. In addition, the two adjustment variables of political skills and perceived organizational support both have a positive impact on the improvement of personal initiative and execution. Therefore, in order to improve the enterprise emergency management ability, it is suggested that enterprises should give full play to the personal initiative and improve the individual and overall executive power. The conclusion of this paper can provide new methodological support for improving emergency management ability.

## Introduction

Emergency management has been attracting attention from all walks of life in recent years (Chen, 2016; Lu and Zhang, 2016; Qin, 2016; Wu and Lei, 2019). At the beginning of 2020, the attack and rapid spread of the COVID-19 virus pushed the topic of enhancing emergency management capabilities to a new hot spot (Fu, 2020; Ma, 2020; Ni et al., 2020; Qiu et al., 2020). In the face of emergencies, under the orderly leadership of the state, effective use of the emergency management ability of the organization can effectively alleviate and contain the spread of adverse events, so it is very important to enhance this ability in daily work (Chang, 2017; Wang, 2018). In the research of various safety accidents and production hazards and other emergency management objects, human factors are always important strategic application points (Qi et al., 2020; Xu, 2020; Zhang, 2020). However, most of the previous research conclusions focused on the measures of safety awareness and professional skills training of training personnel (Wei et al., 2019; Kong et al., 2020; Ren and Nie, 2020), which belong to the category that organizations urge individuals to passively change to meet requirements under the pressure of security assessment and other policies. The role of personal initiative is often overlooked.

Some scholars pointed out that, in the face of a complex and changeable environment, corporate employees are not always passively restricted by them but tend to make breakthroughs and changes, make a series of active behaviors to understand and obtain the information as well as other resources needed for survival and development so as to effectively improve their adaptability to the work and organizational environment (Liu, 2008). At the same time, the modern organizational system and working environment also require employees to have a certain degree of initiative to be able to independently identify and solve problems in the case of completing the established tasks of the post, thereby promoting the smooth completion of the work of the enterprise (Chen and Chen, 2017; Shu, 2020). Campos et al. (2017) showed that, in the context of promoting the development of small businesses in West Africa, the effect of teaching personal initiative is better than that of traditional training. It can be concluded that personal initiative has a certain positive value in organizational activities, guiding people to think whether it can play a positive role in pursuing the improvement of enterprise ability. Through research and summary, personal initiative can increase the effectiveness of individuals and organizations, including enhancing individual creative thinking (Lisbona et al., 2018), improving working conditions in enterprises (Frese et al., 1997), improving job performance (Zhao Y. et al., 2020), etc. Thus, it can be used as an important criterion for the effectiveness of individuals and organizations (Teng, 2014). There are still many studies on personal initiative, but they rarely extend to the mechanism of the relationship between personal initiative and organizational ability.

Although many scholars have carried out in-depth research on the improvement measures and evaluation of personal initiative or enterprise emergency management capability, few scholars have combined these two variables for systematic analysis, nor have they explored the relationship between them and the implicit influence mechanism (Liu, 2008; Geng and Cheng, 2021). But, in real life, there is, indeed, a certain correlation between personal initiative and emergency management ability of enterprise. Individuals with higher initiative can consciously complete their duties, and they tend to actively and effectively provide ideas even when finding problems out of their duty (Teng, 2014). Therefore, companies with highly initiative employees perform their tasks more efficiently. In the face of emergencies, they are able to respond with stronger execution power. The effective exertion of personal initiative can reduce the loss caused by negative coping, ignoring hidden dangers and risks, thereby improving the overall emergency management ability. Thus, this paper introduces executive power as a mediator variable to analyze the direct impact of personal initiative on enterprise emergency management ability and the indirect impact through executive power.

As an individual characteristic, initiative will be affected by external factors. Friction and conflict are not conducive to the full play of initiative (Bolino et al., 2010), which requires individuals to have certain non-technical skills, such as political skills. It helps to adjust behavior in real time in different situations, develop good interpersonal relationships, and alleviate conflicts. Through effective interaction and communication with superiors and colleagues, people will gain recognition and trust (Gao and Ma, 2008), thus speed up the implementation of work and improve the efficiency of overall emergency management. In addition, feature activation theory shows that the activation of individual characteristics is often affected by situational factors. And organizational support perception, as a psychological factor related to individual self-improvement motivation (Kurtessis et al., 2017), can alleviate job burnout and promote active behavior (Yang and Yang, 2020). So this paper also discusses the moderation role of political skills and organizational support perception in the impact model of personal initiative on enterprise emergency management ability.

In summary, this article introduces the concept of executive power and takes the concepts of organizational support theory and political skills as moderating variables, thereby constructing a model of the relationship between personal initiative and corporate emergency management capabilities at the organizational level. Then, SPSS and AMOS software are used for regression analysis and structural equation model analysis to test that model. The aim of this paper is to explore the influence mechanism of individual initiative on the emergency management ability of the enterprise, clarify the influence relationship between internal research variables, and provide some innovative ideas for future emergency work execution and the cultivation and improvement of enterprise emergency management ability.

Starting from individual initiative, this article uses empirical methods to study its impact on the emergency management capabilities of enterprises. We can prove from the data level that personal initiative and emergency management capabilities are, indeed, related so as to provide ideas for talent selection and quality training in organizational management. And through the study of internal mechanisms, we can understand which factors have caused the impact and the actual impact of the impact during the entire process of personal initiative affecting emergency management capabilities. Therefore, we can take corresponding measures to ensure that individual initiative can play its value, thereby promoting the improvement of the emergency management capabilities of the organization.

## Research Theory and Hypotheses

### Personal Initiative and Emergency Management Ability

The concept of personal initiative was first put forward by Fay and Frese (2001). This concept refers to the behavioral pattern of individuals actively overcoming difficulties and obstacles, completing tasks, and achieving established goals. The main characteristics of this personal characteristic include spontaneity, taking the lead in action and overcoming difficulties. Specifically, “spontaneity” refers to that individual who actively takes actions to complete the work tasks that are not clearly required in the organizational responsibilities. “Taking the lead in action” refers to the individual considering problems from a long-term perspective, taking the action in advance to cope with the possible difficulties in the future. “Overcoming difficulties” refers to the courage of the individual to challenge and break through himself or herself, and can actively overcome the problems in the process, constantly approaching and achieving the goal (Cui and Jiang, 2016).

In order to effectively prevent and predict the occurrence of public emergencies to minimize the possible losses or negative impacts caused by public emergencies, the government, departments, units, and other organizations have carried out a series of work, such as the formulation of emergency laws and regulations, emergency plans, and the establishment and improvement of emergency response, namely emergency management (Chen, 2019). Specifically, it refers to the management behaviors taken by various organizations in different stages, such as prediction and prevention, event identification, emergency response, emergency decision-making, disposal, and response evaluation (Yang and Yin, 2019). Emergency management ability refers to the capacity to organize and carry out the above work.

Conceptually, it is obvious that people with initiative, due to their characteristics of “spontaneity,” “taking the lead in action,” and “overcoming difficulties,” may be more likely to adapt to the complex and changeable working environment (Zhao Y. et al., 2020), make appropriate response, and play an active role in all aspects of emergency management. Therefore, from the perspective of an individual level, the improvement of personal initiative can improve the overall emergency management ability of the enterprise.

In addition, according to the social interaction theory, there are behavioral and psychological interactions between individuals, individuals and groups, and between groups. Personal initiative can affect not only oneself but also affect others and even organizations through exchange and cooperation. On the one hand, highly motivated individuals are more willing to share knowledge and conduct organizational learning (Sun and van Emmerik, 2015; Chen and Chen, 2017). Thus, the cognition and understanding of individuals and other members of the organization on work tasks can be effectively improved by promoting the flow of information and knowledge. On the other hand, individuals with initiative are more likely to help others (Zhang et al., 2016), which is conducive to communication and cooperation within the organization. In emergency management, rapid and correct understanding of tasks and good cooperation among organization members can effectively promote the improvement of efficiency. Therefore, from the organizational level, personal initiative can also positively affect the overall emergency management ability of the organization by driving the group effect.

There are also traces to follow in academic research. Ismail et al. (2012) confirmed through the structural equation model that the personal initiative, which occupies the core position in personal factors, can effectively promote the transition of the safety production management mode, and thus can scientifically and effectively improve the ability of organization safety management. Liu (2008) also pointed out that the effective exertion of enthusiasm and initiative of an employee is positively related to the high efficiency and high-quality work, and then has a positive influence on the overall ability of the organization.

According to the above discussion, we speculate that there may be a positive correlation between personal initiative and corporate emergency management ability. Therefore, the first hypothesis of this paper was put forward:

*Hypothesis 1*: personal initiative is positively related to emergency management ability of enterprise.

### Mediating Role of Executive Power

The definition of executive power differs on different levels. At the individual level, it is the ability of individuals to perform their own roles and responsibilities and complete the corresponding tasks, while it refers to the ability of an organization or group to implement and achieve the strategic objectives of the plan at the group level (Yuan, 2012). The strengthening of executive power not only contributes to the management innovation of the organization and the improvement of the comprehensive quality of employees but also effectively promotes the effective operation of the overall management of the enterprise, especially in terms of promoting internal organization coordination (Zhang, 2019). Therefore, there is a certain correlation between the executive power and the management ability at the enterprise level. There are many pieces of research on executive power, which mainly focus on influencing factors, current situation evaluation, strategies, measures, and so on (Cao and Hu, 2020; Gao, 2020; Liu et al., 2020). Although some scholars have used the concept of executive power in the research of influencing mechanism, most of them regard it as an antecedent variable or a result variable (Chen and Zhang, 2019; Zhao K.Y. et al., 2020). Therefore, this paper creatively studies the concept of executive power as the intermediary variable and tries to explore the actual influence relationship between executive power and personal initiative and enterprise emergency management ability.

#### Executive Power and Emergency Management Ability of Enterprises

Some scholars have studied the interaction between executive ability and enterprise-related capabilities, which provides a certain basis for the hypothesis of this paper. Wang (2010) pointed out that the effect of safety management depends on the execution of safety management under certain conditions of production technology, natural conditions, systems, and enterprise culture. Through analyzing and studying the problems existing in the emergency management system, Rao and Liang (2007) pointed out that implementation and executive power are the bases of emergency management. They affirmed that there is a positive relationship between executive power and emergency management ability. From the perspective of conflict management, Zhang et al. (2017) studied the impact of executive power on the level of emergency management of enterprise and verified the relationship model between variables through the structural equation model analysis method. Therefore, we put forward the second hypothesis of this paper:

*Hypothesis 2*: Executive power is positively related to the emergency management ability of enterprises.

#### Executive Power and Personal Initiative

Greenglass and Fiksenbaum (2009) pointed out that individuals with a high level of initiative often work hard to improve their performance and better perform organizational tasks. Taking initiative, the key of intention as the initial motive force has also become a key element in the research of the efficient execution drive mode (Yu, 2019). Above pieces of research show that the executive power of the enterprise can be effectively improved through the cultivation of initiative consciousness and attitude of people. In addition, when studying the execution of emergency management, Rao and Liang (2007) regarded human initiative (including initiative, enthusiasm, creativity, etc.) as one of the important factors of emergency management. It can not only actively predict the execution efficiency of the emergency plan but also determine whether the execution process of the emergency plan is smooth. All of the above studies show that there is a positive relationship between personal initiative and execution power. Therefore, we proposed the following hypothesis:

*Hypothesis 3*: Personal initiative is positively related to executive power.

Combining Hypotheses 2 and 3, the hypothesis that executive power plays a mediating role in the influence model can be formed:

*Hypothesis 4*: executive power mediates the relationship between personal initiative and emergency management ability of enterprise.

### Moderating Role of Political Skills

Ferris et al. (2002) defined the political skills of individuals in the organization as a personal style, including social perception or social acuity, that is, the ability of individuals to adjust their behaviors in different environments or changing circumstances. It can effectively control and influence the behavior of others by triggering trust, self-confidence, and sincerity, and then transform the original “one-person battle” into a mode of joint effort to achieve the goals of an individual or organization faster. According to his multidimensional theoretical model of organizational political skills, political skills not only affect the quality of individuals but also play a role through interpersonal relationships and the team level (Ferris et al., 2007). Employees with high initiative always try to take positive actions to improve the present situation. However, just “making things happen” with a positive personality is not enough. They also need to have the necessary political skills to facilitate “task completion” (Yang et al., 2019). Therefore, this paper intends to introduce the concept of political skills into the influence model, trying to explore whether the effective interaction and communication process between members can positively regulate the relationship between personal initiative and executive power.

Treadway et al. (2013) noted that the political influence process under political skills includes encouraging individual participation and convincing execution, which enables others in the organization to perceive problems according to wishes of the influencer so as to achieve individual and organizational goals. Ferris et al. (2007) also showed that politically skilled individuals achieve their goals through their ability to understand people and the environment. In addition, people with high political skills can accurately evaluate and understand the behavioral expectations in specific situations, thus producing expected responses. Faced with constantly changing expectations and needs, they tend to make adaptive and adjustment behaviors in different situations, and can appropriately calibrate and implement their appropriate behaviors in the context in an effective and influential manner. Adeel et al. (2019) pointed out that political skills can help to reduce the negative attitude toward innovative behavior of subordinates. Even if employees have conflicts with their superiors, their political skills can help them gain positive attitude from their superiors, such as giving support, mobilizing important resources, and sponsoring. Employees with high political skills are also more likely to successfully control social and political processes and achieve creativity (Baer, 2012). In summary, on the one hand, individuals with higher political skills have the ability to understand the current situation, evaluate expected behavior, and respond to risks in complex environments in real time. On the other hand, they are able to influence others and convincingly make them act according to their ideas, which is conducive to achieving team and organizational goals together. In addition, from the perspective of leadership, individuals with higher political skills are able to improve the bad emotions of their superiors and reduce the resistance to the implementation of their own innovative ideas. Once the superiors show positive emotions such as support, individuals will be much smoother and more willing to take the initiative, thus generating a virtuous circle, which is conducive to the realization of goals. It can be concluded that, if individuals have political skills, they can make efforts in a variety of ways to ensure that their initiative works, thereby effectively enhancing execution power. In contrast, it is difficult for individuals with low political skills to really make their initiative work through effective communication with other individuals because of their limited social skills, even if they are willing to promote the achievement of the goals of the organization (Gong, 2019). According to the above discussion, we put forward the corresponding hypothesis:

*Hypothesis 5*: Political skills will positively moderate the relationship between personal initiative and executive ability, and individuals with higher political skills can better play the positive role of their initiative on execution.

### Moderating Role of Perceived Organizational Support

Organizational support theory points out that the benefits employees received from their employers will directly affect the achievement of organizational goals. For example, once employees got more support, they will respond to organizational support through high attendance and punctuality, thus forming more emotional commitments to the organization and responding flexibly to the possible problems in organizational activities (Rhoades and Eisenberger, 2002). According to the principle of reciprocity, if employees realize that they have been rewarded and recognized enough, they will work more efficiently and pay more attention to the achievement of the goals of the organization, thereby enhancing executive power. This leads to the concept of perceived organizational support, as the name implies, the degree of organizational support perceived by individuals. In addition, job performance is the performance of the ability of an individual to achieve a set goal, that is, execution power. Cheng (2019) proved that the degree of individual perceived organizational support can positively predict job performance. Based on the above analysis, this paper speculates that perceived organizational support can effectively affect organizational execution.

On the other hand, in the process of continuous social communication with organizations, the perception of organizational support plays an important role in determining the psychological cognition of employees, and is an important driving force for individuals to make positive work behaviors. In order to change the work cognition and attitude of the individuals, the most important thing is to make them feel that the pay and return are equal (Wang and Zhao, 2020). Fay and Frese (2001) pointed out that environmental supports can stimulate self-started behavior, and then confirmed the correlation between individual perceived psychological security and personal initiative. Many scholars have also studied the relationship between perceived organizational support and individual specific behavioral outcomes. Wang and Zhao (2020) proved that high organizational support perception can convey information that the organization is willing to give back to employees in the form of rewards, so they usually make more positive behaviors and additional role behaviors accordingly. At the same time, the perception of organizational support often enhances sense of identity and belonging of employees to the organization so as to promote individuals to actively take positive actions and contribute to the development of the organization. They are more willing to put forward their own opinions in the face of old rules and dogmas that do not adapt to the actual situation. What is more, they are willing to make more active attempts to solve problems. Eisenberger et al. (2020) also pointed out that employees with high organizational support perception are more likely to talk about security issues and show support for new technologies. Innovative behavior, problem prevention, suggestion behavior, etc., corresponding to the research variables mentioned in the above research, are all active behaviors of employees, and the performance of personal initiative. It shows that perceived organizational support is closely related to personal initiative and may trigger personal initiative to a certain extent.

Based on the above analysis of the relationship among perceived organizational support, executive power and personal initiative, we speculate that perceived organizational support may be able to moderate the relationship between personal initiative and executive power. When individuals gain a high level of perceived organizational support, it can promote their initiative and enhance executive power. Therefore, the following assumptions are put forward:

*Hypothesis 6*: Perceived organizational support will positively moderate the relationship between personal initiative and executive power. Compared with low perception individuals, high perceived individuals can exert the positive influence of their initiative on executive power better.

**FIGURE 1:**
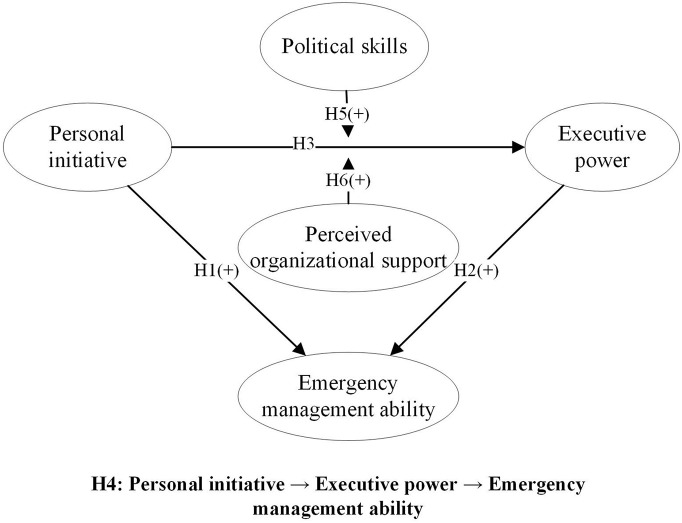
Research structure.

Based on the above discussion, the theoretical model of this study is formed, as shown in Figure 1.

## Materials and Methods

### Participants and Procedure

#### Participants

The participants are full-time employees of companies in high-risk industries, such as chemical industry, construction, and transportation. These companies are located in Jiangsu, Zhejiang, Shanghai, and other places in China with rapid economic development.

Of the entire sample, 40% of the participants were male and 60% were female. The ages of participants were divided into different age groups as follows: under age 25 (67.79%), 26–35 years old (21.63%), 36–45 years old (5.77%), and 46 years old and above (4.81%). Regarding the education level of the participants, 2.29% of them have junior high school education and below, 1.83% of them have high school education, 7.8% of them have junior college degree, 79.36% of them have bachelor degree, 8.72% of them have master’s degree or above. In terms of enterprise type, which the sample personnel engaged in, state-owned enterprises account for 18.35%, private/private enterprises account for 46.79%, foreign-funded enterprises (including wholly foreign-owned, Sino-foreign joint ventures, Sino-foreign cooperation) account for 6.42%, social organizations account for 5.5%, and others account for 22.94%. In terms of the position of the sample personnel in the enterprise, the grassroots employees account for 75.63%, while the managers account for 24.37%. With regard to job tenure, 56.42% have worked for their organizations for less than 1 year, 28.44% for 1–3 years, 5% for 3–5 years, 6.42% for 5–10 years, and 3.22% for more than 10 years. Overall, the distribution of samples is diverse.

#### Procedure

Due to the impact of COVID-19 virus, data collection depends on an online questionnaire survey. First of all, we contacted classmates, friends, and relatives working in chemical, construction, transportation, and other industries in different cities. After their consent, we posted the online questionnaire in their company groups *via* Wechat and QQ. The participants in this study voluntarily participated by filling out online questionnaires, so the data obtained are random. Moreover, in order to ensure the effectiveness of the questionnaire, the filling time of the questionnaire is controlled within 20 min. The entire questionnaire process lasted approximately 1 month, from April to May 2020. A total of 270 questionnaires were returned. After removing the questionnaires with obvious regularity and consistency, 208 valid samples were finally collected (the recovery rate was 77%).

#### Measures

For the measurement of variables, this paper refers to the validated scales in the existing Chinese and English literature to collect relevant data. So all scales have good reliability and validity. In order to ensure the accuracy of wording, English scales were translated into Chinese by professionals. During data collection, items of all variables were measured on a five-point Likert scale (1 = strongly disagree, 5 = strongly agree).

##### Personal Initiative

Personal initiative was measured with a five-item scale, which was modified according to Chinese cultural background based on the scale developed and verified by Frese et al. (1997). Sample items include: “I will seize the opportunity quickly to achieve my goal,” and “I usually make some preparations in advance to achieve a certain goal.”

##### Political Skills

According to the measurement scale of individual political skills proposed by Ferris et al. (2005), five-item scales were formed for this study. Examples of statements are “I will use my interpersonal skills at work to influence others” and “I am good at using relationships to ensure the completion of tasks.”

##### Perceived Organizational Support

We used the five-item scale proposed by Eisenberger et al. (2001) to measure the degree to which individuals perceive organizational support. Sample items include: “Business is willing to help when I need it” and “Business values my efforts.”

##### Executive Power

As for the executive power measurement scale, we selected the five-item scale of enterprise-level executive power improved by Zhou (2018). He developed this questionnaire based on the existing questionnaire and verified its effectiveness through research. Examples of items of this scale are “The company’s strategic initiatives can be implemented” and “In the course of execution, the plan can always be adjusted according to the situation.”

##### Emergency Management Ability of Enterprise

As for the measurement of emergency management ability, there is no recognized and unified scale that can be directly used in this study. We referred to the questionnaire used by Wang (2015) in evaluating the emergency management ability of petrochemical enterprises and formed the seven-item scale after sentence adjustment. Sample items include: “In emergency management, enterprises can quickly analyze and make decisions, and then organize accident site coordination and allocate emergency resources reasonably” and “After the accident, the enterprise can carry out the emergency recovery work in time.”

##### Control Variables

In order to exclude the potential impact of demographics and some variables related to work background in this study, we controlled for six variables: gender, age, education level, nature of enterprise, position, and work tenure. Each of these variables is frequently included in relevant studies (Cui and Jiang, 2016; Fu, 2020). First, gender was coded as 0 for male and 1 for female. Second, age was coded as 1 for“≤25,” 2 for “26–35,” 3 for “36–45,” 4 for “>46.” Third, the education level was coded as 1 for “junior high school and below,” 2 for “high school,” 3 for “junior college,” 4 for “undergraduate,” 5 for “master’s degree or above.” Fourth, nature of enterprise was coded as 1 for “state-owned enterprises,” 2 for “private/private enterprises,” 3 for “foreign-funded enterprises,” 4 for “social organizations,” 5 for “others.” Fifth, position was coded as 1 for “the grassroots employees” and 2 for “managers.” Finally, work tenure was coded as 1 for “less than 1 year,” 2 for “1–3 years,” 3 for “3–5 years,” 4 for “5–10 years,” and 5 for “more than 10 years.”

### Analysis Strategy

In the part of data analysis and hypothesis testing, we firstly used SPSS 22.0 software to test the reliability and common method deviation of the collected data. And the mean value, standard deviation, and correlation coefficient of all variables were measured. Secondly, we conducted a confirmatory factor analysis (CFA) using AMOS software to examine the validity of structure, convergence, and distinction (Cheung and Wong, 2011; Choi and Moon, 2017). Lastly, through the structural equation model analysis, the bootstrap method of process program and the regression analysis, we verified and analyzed the hypotheses of this paper.

In order to verify the validity of the estimated model, we selected χ^2^/df (Chi square degree of freedom ratio), root mean square error of approximation (RMSEA), goodness-of-fit index (GFI), comparative fit index (CFI), and normed fit index (NFI) to test the fit of the model. It is acceptable for χ^2^/df to be between 1 and 5 (Salisbury et al., 2002). The CFI, NFI, and GFI should be over 0.90 (Salisbury et al., 2002), and the value of RMSEA should be less than 0.08 (Byrne, 2006).

## Results

### Reliability Analysis

Firstly, the reliability of the scale used in this paper was tested by SPSS 22.0 software. Statistics on the Cronbach’s Alpha of different scales and the overall questionnaires are shown in Table 1. It can be seen from Table 1 that the minimum Cronbach’s Alpha of each variable is 0.870, and the maximum is 0.930, which is greater than 0.7, and the overall Cronbach’s Alpha is more than 0.9, indicating that all the scales have good reliability.

**TABLE 1:** Statistics of Cronbach’s α coefficient.

Latent variable	Cronbach’s α	
Personal initiative	0.889	0.941
Political skills	0.870	
Perceived organizational support	0.879	
Executive power	0.895	
Emergency management ability	0.930	

### Common Method Deviation Test

First of all, Harman single factor test was used to test the common method deviation. The results show that the overall KMO value is 0.936, and five factors with eigenvalues greater than 1 are selected eventually. The total contribution rate reached 70.084%; the interpretation contribution rate of the first factor before rotation was 39.793% (less than 40%). In addition, in the later confirmatory factor analysis, it can be seen that the fitting effect of the single factor model is significantly worse than that of the model established in this paper, so there is no serious common method deviation in this study.

### Validity Analysis

#### Convergent Validity

In this part, AMOS software was used for confirmatory factor analysis to analyze the convergent validity of the whole theoretical model. The factor loads of all constructs are mainly considered in the evaluation of convergence validity. The following table (see Table 2)shows the statistics of factor loads of each path of the model.

**TABLE 2:** Factor load statistics.

Path			Estimate	AVE	CR
PI5	<—	PI	0.846	0.639	0.898
PI4	<—	PI	0.8766		
PI3	<—	PI	0.7631		
PI2	<—	PI	0.7486		
PI1	<—	PI	0.7551		
PS5	<—	PS	0.7657	0.573	0.870
PS4	<—	PS	0.7569		
PS3	<—	PS	0.713		
PS2	<—	PS	0.7445		
PS1	<—	PS	0.8026		
POS5	<—	POS	0.7457	0.600	0.882
POS4	<—	POS	0.7276		
POS3	<—	POS	0.7993		
POS2	<—	POS	0.8279		
POS1	<—	POS	0.7692		
EP5	<—	EP	0.8257	0.634	0.896
EP4	<—	EP	0.8071		
EP3	<—	EP	0.8171		
EP2	<—	EP	0.7768		
EP1	<—	EP	0.7518		
EMA5	<—	EMA	0.8491	0.639	0.925
EMA4	<—	EMA	0.8068		
EMA3	<—	EMA	0.8245		
EMA2	<—	EMA	0.8199		
EMA1	<—	EMA	0.7244		
EMA6	<—	EMA	0.8055		
EMA7	<—	EMA	0.7601		

*PI, personal initiative; PS, political skills; POS, perceived organizational support; EP, executive power; EMA, emergency management ability.*

*PI1-PI5, PS1-PS5, POS1-POS5, EP1-EP5, and EMA1-EMA7 scale items corresponding to the above study variables.*

It can be seen from Table 2 that the factor load of each path is greater than 0.7, which is significant at the level of *p* = 0.001, indicating that there is good internal consistency between the items of the scale and the latent variables described. In addition, according to the established formula, the average extraction variance (AVE) of each latent variable is greater than 0.5, and the combined reliability (CR) is greater than 0.7, so the whole model has good convergence validity.

#### Discriminant Validity

In this paper, confirmatory factor analysis was used to test the discriminant validity. From the model fitting results in Table 3, it can be seen that the fitting indexes of the theoretical model used in this paper all meet the standard. Among them, Chi-square value is 315.062, degree of freedom is 309, and χ^2/^df is 1.02 and is in the range of 1–5. Furthermore, RMSEA is 0.01, less than 0.05. GFI is 0.901, NFI is 0.917, and CFI is 0.998, greater than 0.9, indicating that the model has good fitting effect and structural validity. In addition, through comparing the data of fitting index with those of the other nested models, it can be seen that the theoretical model used in this study is significantly better, indicating that the theoretical model has good discriminant validity.

**TABLE 3:** Fitting results of the confirmatory factor analysis model.

Model	Factors contained	χ*^2^*	DF	RMSEA	GFI	NFI	CFI
Five factor model	PI, PS, POS, EP, EMA	315.062	309	0.010	0.901	0.917	0.998
Four factor model	PI + PS, POS, EP, EMA	402.858	311	0.038	0.876	0.894	0.973
Four factor model	PI, PS + POS, EP, EMA	517.946	315	0.056	0.841	0.863	0.941
Four factor model	PI, PS, POS + EP, EMA	620.671	318	0.068	0.770	0.836	0.912
Four factor model	PI, PS, POS, EP + EMA	680.371	318	0.074	0.746	0.820	0.894
Three factor model	PI + PS + POS, EP, EMA	945.288	321	0.097	0.667	0.750	0.818
Three factor model	PI, PS + POS + EP, EMA	925.331	321	0.095	0.669	0.755	0.824
Three factor model	PI, PS, POS + EP + EMA	977.167	321	0.099	0.645	0.741	0.809
Two factor model	PI + PS + POS + EP, EMA	1,260.699	323	0.118	0.586	0.667	0.727
Two factor model	PI, PS + POS + EP + EMA	1,304.401	323	0.121	0.568	0.655	0.714
Single factor model	PI + PS + POS + EP + EMA	1,686.260	324	0.143	0.497	0.554	0.603

*PI, personal initiative; PS, political skills; POS, perceived organizational support; EP, executive power; EMA, emergency management ability.*

According to the above analysis of the reliability and validity of the model, each part has passed the test, indicating that the measurement model meets the requirements.

### Descriptive Statistical Analysis

This part lists the basic statistical information and correlation coefficient of control variables and research variables. From Table 4, it can be seen that the correlation between personal initiative, political skills, perceived organizational support, executive power, and emergency management ability has reached a significant level (*p* < 0.01). To some extent, the Hypothesis 1–3 proposed in this paper is also confirmed. In addition, the correlation between the control variables and the five research variables can be observed. Age is positively correlated with personal initiative and perceived organizational support (*p* < 0.05). Positions show significant positive correlation with personal initiative, political skills, executive ability, and emergency management ability at different levels. Job tenure is also positively correlated with the five research variables in this paper. There is no correlation between the two control variables of gender as well as the education level and the research variables in this study.

**TABLE 4:** Mean value, standard deviation, and correlation coefficient of main variables.

	M	SD	Q1	Q2	Q3	Q4	Q5	Q6	PI	PS	POS	EP	EMA
Q1	1.66	0.033	1										
Q2	1.47	0.056	−0.157*	1									
Q3	3.90	0.046	−0.026	−0.412**	1								
Q4	2.66	0.100	0.138*	−0.156**	0.026	1							
Q5	1.25	0.030	0.004	0.240**	−0.069	0.009	1						
Q6	1.70	0.072	−0.087	0.587**	−0.277*	−0.199*	0.303**	1					
PI	4.00	0.039	−0.100	0.169*	−0.053	−0.126	0.204**	0.284**	1				
PS	3.76	0.039	−0.021	0.065	−0.039	−0.190*	0.171*	0.142*	0.498**	1			
POS	3.64	0.039	−0.031	0.169*	−0.031	−0.087	0.160*	0.213**	0.396**	0.479**	1		
EP	3.78	0.038	0.016	0.035	−0.018	−0.040	0.093	0.150*	0.393**	0.382**	0.569**	1	
EMA	3.72	0.040	0.096	0.018	−0.094	0.002	0.169*	0.192**	0.361**	0.423**	0.495**	0.605**	1

Valid *N* = 208, M as average, SD as standard deviation *p < 0.05; **p < 0.01.

*Q1–Q6 represent six control variables: gender, age, education level, enterprise type, position, and job tenure; PI, personal initiative; PS, political skills; POS, perceived organizational support; EP, executive power; EMA, emergency management ability.*

### Mediating Effect Tests

**FIGURE 2:**
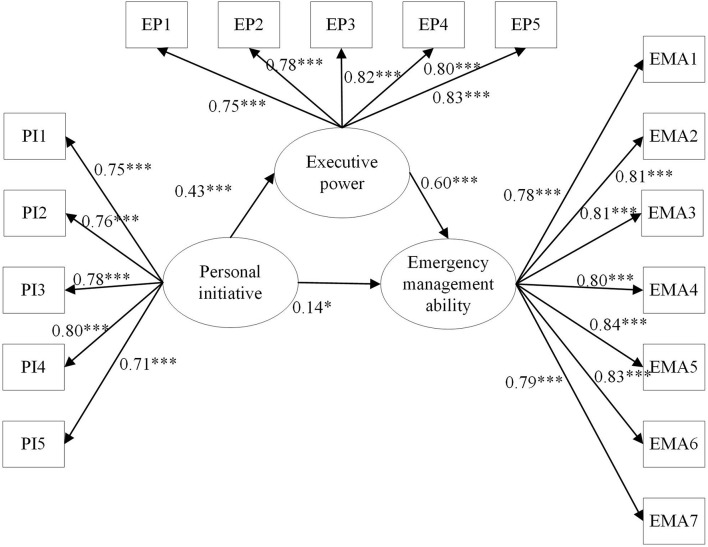
Mediating effect model PI1 PI2, PI3, PI4, and PI5 are five observation variables of personal initiative, EP1, EP2, EP3, EP4 and EP5 are five observation variables of executive power, EMAI, EMA2, EMA3, EMA4, EMAS, EMA6, and EMA7 are seven observation variables of influence emergency management ability of enterprise ^∗^*p* < 0.05, ^∗∗^*p* < 0.01, and ^∗∗∗^*p* < 0.001.

In this paper, we used the structural equation model analysis method to test the hypotheses. The mediating effect model used in the verification process is shown in Figure 2 below. According to the fitting index of the model (see Table 5), the chi-square degree of freedom ratio and RMSEA, GFI, NFI, and CFI were selected. The chi square degree of freedom ratio of the model is less than 2, RMSEA is equal to 0.05, and GFI, NFI, and CFI are all greater than 0.9, which are within the standard of judgment, indicating that the model fits very well.

**TABLE 5:** Model fit indicators.

Fitting index	χ*^2^/df*	RMSEA	GFI	NFI	CFI
Numerical value	1.511	0.050	0.912	0.928	0.974
Standard	1–3	0.05–0.08	>0.9	>0.9	>0.9

It can be seen from the path inspection statistical table (Table 6) that the path between the studied variables in the mediation model is significant, which validates Hypotheses 1–3. Among them, personal initiative has a positive impact on execution power, and the standardized path coefficient is 0.4317 (*p* < 0.001), showing that Hypothesis 1 is tenable. Executive power has a positive impact on the emergency management level and the standardized path coefficient is 0.5961 (*p* < 0.001), so Hypothesis 2 is supported. What is more, personal initiative positively affects the emergency management level, and the standardized path coefficient is 0.1401 (*p* < 0.05); thus, Hypothesis 3 is also tenable.

**TABLE 6:** Path test statistics.

	Standardization coefficient	S.E.	C.R.	*P*
PI → EP	0.4317	0.0732	5.6559	***
PI → EMA	0.1401	0.0795	2.0323	*
EP → EMA	0.5961	0.0932	7.6849	***

*PI, personal initiative; EP, executive power; EMA, emergency management ability.*

***p < 0.001; *p < 0.05.

In addition, the deviation correction percentile bootstrap method was used to test the mediating effect through AMOS software. In this study, 2,000 repeated samples are selected (*N* = 208). The test results are shown in Table 7 below, *P* value is 0.0007, and the 95% partial correction confidence interval is (0.1656, 0.4686), excluding 0, so the mediating effect is significant, indicating that Hypothesis 4 holds. In addition, the standardized effect value can be obtained according to the output of AMOS, in which the direct effect of personal initiative on the execution power is 0.4317, and the direct effect value of execution power on emergency management ability is 0.5961. The total effect of personal initiative on emergency management ability is 0.3974, of which the direct effect is 0.1401, accounting for 35.3% of the overall effect. Accordingly, the indirect effect is 0.2573, accounting for 64.7% of all. In contrast, the indirect effect formed by mediating variables can better constitute a positive correlation between personal initiative and enterprise emergency management ability, which confirms the importance of the mediating effect of execution power in the model.

**TABLE 7:** Mediating Effect Bootstrap test (non-standardized coefficient).

route	Effect value	SE	Bias-corrected			Percentile		
			95% CI			95% CI		
				
			Lower	Upper	*P*	Lower	Upper	*P*
PI→EP →EMA	0.2969	0.0771	0.1656	0.4686	0.0007	0.1594	0.459	0.001

*PI, personal initiative; EP, executive power; EMA, emergency management ability.*

### Moderating Effect Tests

**FIGURE 3:**
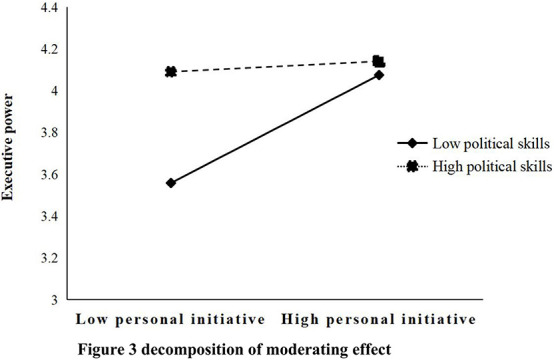
Decomposition of moderating effect.

The moderating effect test in this study mainly adopted the hierarchical regression analysis and the bootstrap of process proceedings through SPSS software. In the regression analysis, the control variables were placed on the first page, the personal initiative and execution were placed on the second page, and the interaction item was placed on the third page. Finally, according to the output results of hierarchical regression analysis, the moderating effect test table of political skills and perceived organizational support (see Tables 8, 9) were drawn. And the moderating effect decomposition diagram (see Figure 3) was also drawn according to the output results of the bootstrap method of the process program. Therefore, it is easy to intuitively understand the moderating role of moderating variables between personal initiative and execution power.

**TABLE 8:** Moderating effect test of political skills.

Model 1				Model 2		
	Standardization coefficient	T	Significance	Standardization coefficient	T	Significance
	Beta			Beta		
Gender	0.04	0.614	0.54	0.051	0.794	0.428
Age	–0.062	–0.737	0.462	–0.063	–0.765	0.445
Education level	0.005	0.078	0.938	–0.013	–0.194	0.846
Enterprise type	0.046	0.693	0.489	0.051	0.784	0.434
Position	–0.019	–0.28	0.78	–0.016	–0.24	0.81
Job tenure	0.096	1.164	0.246	0.121	1.482	0.14
Personal initiative	0.263	3.479	0.001	0.247	3.298	0.001
Political skills	0.255	3.438	0.001	0.316	4.099	0.000
Interaction item				–0.17	–2.514	0.013
R^2^	0.21			0.234		
F	6.598**			6.724**		

**p < 0.01.

**TABLE 9:** Moderating effect test of perceived organizational support.

Model 1				Model 2		
	Standardization coefficient	T	Significance	Standardization coefficient	T	Significance
	Beta			Beta		
Gender	0.036	0.615	0.539	0.042	0.731	0.466
Age	–0.115	–1.53	0.128	–0.113	–1.514	0.132
Education level	–0.023	–0.374	0.709	–0.034	–0.54	0.589
Enterprise type	0.02	0.342	0.732	0.021	0.359	0.72
Position	–0.022	–0.36	0.719	–0.028	–0.471	0.638
Job tenure	0.06	0.814	0.417	0.076	1.015	0.311
Personal initiative	0.207	3.249	0.001	0.207	3.26	0.001
Perceived organizational support	0.499	8.039	0.000	0.513	8.152	0.000
Interaction item				–0.077	–1.282	0.201
R square	0.368			0.373		
F	14.48**			13.096**		

**p < 0.01.

### The Moderating Effect Test of Political Skills

Table 8 shows the moderating effect of political skills between personal initiative and emergency management ability. In the table, the relevant data of two models in the output of regression analysis through SPSS were selected. The first model was the influence model of control variables, personal initiative and political skills on executive power. The second model added the interaction items after decentralization of the two variables of personal initiative and political skills on the basis of the first model. Obviously, the F test of the two models is significant at the level of *p* = 0.01. Comparing the R square values of the two models, it is obvious that the R square value increases by 0.024 after adding the interaction item. In addition, in Model 2, the interaction item after decentralization of the two variables of personal initiative and political skills is *T* tested significant with regression coefficient of −0.17 (*p* < 0.05), indicating that political skill has significant moderating effect on the relationship between personal initiative and executive power, but it weakens the influence of personal initiative on executive power to some extent. The specific regulation effect can be seen vividly in the following decomposition diagram.

This study used the point selection method and drew the moderating effect decomposition diagram according to the output result of bootstrap of process proceedings, which directly shows the difference of the regulation effect between the relationship among personal initiative and emergency management ability under the level of high political skills (one standard deviation above the average) and a low level of political skills (one standard deviation below the average). From Figure 3, the initiative shown by individuals with low political skills can significantly improve executive power. Although the initiative of high-political-skill individuals still has a certain positive impact on executive ability, it is significantly weakened (with a smaller slope), indicating that political skills have a weakening moderating role between personal initiative and emergency management ability of enterprise, which is inconsistent with Hypothesis 5 of this study, so Hypothesis 5 is not tenable.

### The Moderating Effect Test of Perceived Organizational Support

Table 9 also shows the data on the moderating effect of perceived organizational support between personal initiative and executive power in regression analysis. Obviously, the *T* test of the interaction item after decentralization of the two variables of personal initiative and perceived organizational support is not significant (*P* = 0.201 > 0.05). Therefore, the moderating effect of perceived organizational support between personal initiative and executive power is not significant. Hypothesis 6 set in this study is not tenable.

## Discussion

The Hypotheses 1–4 in this study were verified to be true, which confirms the positive effect of personal initiative on executive power as well as enterprise emergency management ability, and the intermediary effect of executive power in this model is also proved. It is clear that enhancing the personal initiative can effectively improve the executive power and enhance the emergency management ability of enterprises through direct and indirect methods.

Through hypothesis testing, Hypothesis 5 in this paper was proved to be not tenable. Although political skills have a significant moderating effect between personal initiative and executive power, it presents a weakening effect contrary to the assumption, that is, individuals with high political skills will weaken the positive impact of their initiative on executive power. There may be several reasons for this:

(1) Individuals with high political skills will have a sense of fatigue when they constantly adjust their behavior, thereby weakening the positive impact of personal initiative on executive power (Tan and Kong, 2020). In organizational life, individuals with higher political skills can more effectively identify and use different information continuously sent by others and their surroundings. And they can adjust their behavior in real time according to the changing environmental needs (Bolino et al., 2010). However, personal initiative may not always exert positive effects, or it may not produce positive effects in a short period of time. Individuals with high political skills in a complex political environment may choose to filter out the tendency of such behaviors due to lack of professionalism such as low sense of accomplishment (Wei and An, 2020), resulting in the weakening of the positive effect of personal initiative on executive power.
(2) The influence of personal initiative on executive power may be more reflected in individual professional ability and technology. Political skills, as a kind of social skills, cannot fully exert their positive influence, support, and recognition on others in the long term and complex organizational political life. Instead, individuals are committed to acquiring certain organizational rights in the process of obtaining resources and achieving goals, which, in turn, poses a status threat and tensions to leadership (Liu et al., 2018). In addition, the initiative shown by individuals may be the same as other personality characteristics, which may easily lead to jealousy or exclusion between mature leaders and numb colleagues, and then cause certain resistance to the enhancement of executive power, which requires group and team cooperation and coordination between the upper and lower levels (Ye et al., 2020).
(3) About 84.86% of the subjects in this study have worked for 3 years or less, and the proportion of grassroots employees is large. Maybe, the political skills of these individuals are not perfect, or the role of political skills in communication and interaction among groups is not fully played due to the short working years, job restrictions, and the influence of the organizational environment and others.

In addition, the moderating effect of perceived organizational support between personal initiative and executive power in Hypothesis 6 was also proved to be insignificant in hypothesis testing. The following three reasons may explain for it.

(1) Although individuals with high perception of organizational support can reduce their concerns about risks and make them more motivated to play their own initiative, there are always some people in the entire organization who are afraid of risks and choose to give up at different times, such as early or midterm. The process may involve individual trait differences similar to risk-taking traits, that is, personal risk-taking or risk-avoiding tendencies (Sun et al., 2018; Sun and Zhao, 2019; Yang, 2020). If the correct behaviors, creativity, ideas, etc., which were derived from personal initiative, cannot be expanded and recognized in the organizational unit, it will not be able to give full play to the positive role of execution power.
(2) Individuals in the organization are not faced with a simple personal situation but a group context in which individuals, other individuals, and the organization interact with one other. Therefore, whether the motivating effect of organizational support can be played effectively depends not only on one’s own perception but also on social communication (Liu et al., 2015). Individuals compare their situations with those of others, and differences in the degree of organizational support perception that individuals and others receive may trigger a sense of injustice. On the one hand, it is not conducive to the coordination within the organization and the full play of personal ability. On the other hand, perceived injustice will prevent the social communication between employees and the company from completing normally. Therefore, the perceived organizational support cannot play a positive role in moderating the relationship between personal initiative and execution power.
(3) The average value of perceived organizational support in this sample is lower than the average of other research variables. This may be due to the fact that most of the sample objects have been employed for less than 3 years or the exploitative nature of enterprises; thus, the individuals do not get enough support they need, resulting in the negative regulatory effect of perceived organizational support personal initiative on executive power.

Although political skills and perceived organizational support have not been proved to play a moderating role in the hypothesis between personal initiative and executive power, there is no doubt that these two variables are positively correlated with personal initiative and executive power. By cultivating political skills and enhancing the perception of organizational support, the interaction and cooperation between individuals and other members of the organization can be strengthened. On the other hand, it will encourage individuals to exert their initiative when they realize that they have received spiritual and practical material support. Generally speaking, the analysis of these two elements has certain research significance for improving the emergency management ability of enterprises.

### Theoretical Implications

This study mainly has three theoretical contributions. Firstly, the existing studies either focus on the cultivation of personal initiative and consequence analysis, or the analysis of influencing factors and the construction of the evaluation model for emergency management ability. Few studies connect the two variables, let alone combine them into a theoretical model for empirical analysis. This paper combines personal initiative with emergency management ability, and then to research and analysis the influence mechanism between them. It fills in the application and research of the concept of personal initiative in emergency work. On the other hand, it enriches the research on the consequences of personal initiative in the aspect of enterprise capability. Secondly, the concept of executive power is introduced into the model as an intermediary variable, which expands the influence of personal initiative on the emergency management capability of the enterprise in the indirect path. This further deepens the correlation between personal initiative and emergency management ability. Finally, we believe that, in the process of the transition from personal initiative at the individual level to executive power at the organizational level, there is interaction, communication, and cooperation among organizational members. Therefore, we explore whether the two potential variables of political skills and organizational support play the role of regulatory variables in the model and their actual role. It is helpful to systematically analyze how personal initiative plays a positive role in emergency work scenarios.

### Practical Implications

Our results also have several important practical implications. First, they confirm that improving personal initiative can effectively improve the emergency management ability of enterprises. Due to the difference between innate and acquired in the acquisition of this ability (Teng, 2014), the effective use of this feature should also be considered from two angles. On the one hand, for employees with innate personal initiative, the most important thing is that the organizations should arrange them in a position that can give full play to their initiative and give them certain rights and freedom. On the other hand, by establishing a reasonable incentive mechanism, fully stimulating individual motivation, establishing person post-matching evaluation and responsibility system, and setting up a certain pressure situation, it can drive individual enthusiasm and sense of responsibility, which is conducive to inducing individual initiative.

Second, we find that the impact of personal initiative on enterprise emergency management ability is also reflected in the indirect path of execution, so the improvement of execution power is also very important. In addition to the introduction of information resources and advanced technology by enterprises, organizations should pay attention to the gradual progress when setting goals, adhere to the process of accumulating small goals to achieve large goals, pay attention to the differences among different people when setting goals and not assign complicated tasks to careless people. On the other hand, it is for individual cultivation and training. In addition to training in professional skills and safety skills guidance, regular safety drills and emergency evacuation should also be used to cultivate and enhance individual response capabilities and team cohesion (Wang, 2010, Wang, 2012).

Finally, through the research, although the positive relationship between personal initiative and executive power is not changed, we find that the positive impact of the initiative of individuals with high-level political skills on executive power is reduced. Therefore, enterprises should be cautious in the work arrangement of relevant personnel in order to maximize the positive role of personal initiative. It cannot be ignored that political skills and perceived organizational support have a positive effect on the improvement of individual initiative and execution. So enterprises should adopt effective methods, such as systematic practice, alternative learning, and communication skills training, to cultivate individual political skills (Gao and Ma, 2008). It is also important to cultivate unity and team awareness through training, team building, and other organizational activities. In addition, enterprises should provide a good working environment, fair competition, and promotion opportunities as well as other resources.

### Limitations and Future Research

The original hypotheses of this study have not been fully verified, so there must be deficiencies and limitations. The shortcomings of this research and the prospects for future research are summarized as follows:

(1) Some scales used in this study refer to the foreign scales, but there are still differences between these scales and the local scales in China. Therefore, we should further explore and develop local scales.
(2) In terms of sample collection, although a variety of options are set up on the basis of the six control variables, most of them are still concentrated on individual options, such as the age of 25 and below, and the education level of undergraduate, which may have a certain adverse impact on the verification of research hypotheses. In the future, methods should be selected to investigate the sample population of other options to enrich the diversity of samples.
(3) This study adopted the method of horizontal research. So longitudinal data should be collected to further demonstrate the relationship between variables after time.
(4) There is a transition from the individual level to the organization level between personal initiative and emergency management ability of enterprises. It may not be comprehensive to describe the influence mechanism of these two variables simply based only on the executive power, political skills, and perceived organizational support proposed in this study. Future research should open up new ideas, carefully study the complex relationships between individuals in the organization, clarify the influence relationships between various elements in the organization, and enrich the model by adding different variables and theories so as to deeply study the influence mechanism of personal initiative on emergency management ability of enterprise.

## Conclusion

Starting from personal initiative, this paper analyzes the influence mechanism between personal initiative and emergency management ability. According to our research results, the improvement of personal initiative helps to improve the enterprise emergency management ability, and it can further positively affect the emergency management ability by indirectly improving the execution ability. Therefore, in high-risk industries, we should pay attention to the cultivation and promotion of personal initiative in emergency management. In addition, political skills have a negative regulatory effect on the relationship between personal initiative and executive power (without changing the positive correlation between them). Moreover, both political skills and perceived organizational support have a positive relationship with personal initiative and execution, so their positive role should also be carefully and appropriately used. This study combines personal initiative and emergency management ability to carry out empirical analysis. Thus, it enriches the research on emergency theme and provides enlightenment and suggestions for the construction of enterprise emergency management ability.

## Data Availability Statement

The original contributions presented in the study are included in the article/supplementary material, further inquiries can be directed to the corresponding author/s.

## Ethics Statement

The studies involving human participants were reviewed and approved by the Ethics Committee of China University of Mining and Technology. The patients/participants provided their written informed consent to participate in this study in accordance with the Declaration of Helsinki.

## Author Contributions

SL conducted the overall control and guidance for this study. FX designed and drafted the manuscript, and then collected and analyzed the data. ZX provided guidance in the process. YK and QY participated in the distribution of the questionnaires and the modification and improvement of the manuscript. All authors contributed to the article and approved the submitted version.

## Conflict of Interest

The authors declare that the research was conducted in the absence of any commercial or financial relationships that could be construed as a potential conflict of interest.

## Publisher’s Note

All claims expressed in this article are solely those of the authors and do not necessarily represent those of their affiliated organizations, or those of the publisher, the editors and the reviewers. Any product that may be evaluated in this article, or claim that may be made by its manufacturer, is not guaranteed or endorsed by the publisher.

## References

[B1] Adeel A. Zhang P. C. Saleem F. Ali R. Batool S. (2019). Conflicts and creative idea endorsement: Do subordinates’ political skills and implementation instrumentality matter? *Int. J. Conflict Manage.* 30 637–656. 10.1108/IJCMA-02-2019-0033

[B2] Baer M. (2012). Putting creativity to work: the implementation of creative ideas in organizations. *Acad. Manage. J.* 55 1102–1119. 10.5465/amj.2009.0470

[B3] Bolino M. Valcea S. Harvey J. (2010). Employee, manage thyself: The potentially negative implications of expecting employees to behave proactively. *J. Occupat. Organizat.* 83 325–345. 10.1348/096317910X493134

[B4] Byrne B. M. (2006). *Structural Equation Modeling with EQS: Basic Concepts, Applications, and Programming.* New York, NY: Routledge.

[B5] Campos F. Frese M. Goldstein M. Iacovone L. Johnson H. C. Mckenzie D. (2017). Teaching personal initiative beats traditional training in boosting small business in West Africa. *Science* 357 1287–1290. 10.1126/science.aan5329 28935805

[B6] Cao G. H. Hu L. L. (2020). Influencing factors and promotion paths of grassroots government execution. *J. South China Univers. Technol.* 22 115–124.

[B7] Chang L. Y. (2017). Significance of emergency management in petrochemical industry. *Chem. Enterprise Manage.* 20.

[B8] Chen G. Q. Chen Z. D. (2017). Empirical study onimpactof proactive personality on individuallearning capability. *J. Technol. Econom.* 36 38–45.

[B9] Chen H. M. (2019). Development of emergency management in China. *China Petroleum Chem. Standard Qual.* 39 99–100.

[B10] Chen X. P. (2016). A review of the literature on public emergency management. *China Manage. Informat.* 19 236–239.

[B11] Chen Y. J. Zhang X. J. (2019). The influence of grass-roots civil servants’ policy execution to the honest behavior of government affairs. *Administrat. Law* 2019 58–67.

[B12] Cheng C. (2019). Research on the relationship between perceived organizational support and job performance—the mediating effect of workplace well-being. *J. Lanzhou Univer. Finance Econom.* 35 97–106.

[B13] Cheung M. F. Wong C. S. (2011). Transformational leadership, leader support, and employee creativity. *Leader. Organ. Dev. J.* 32 656–672. 10.1108/01437731111169988

[B14] Choi B. K. Moon H. K. M. (2017). Subordinates’ helping, voice, and supervisors’ evaluation of job performance: the moderating effects of supervisor-attributed motives. *Career Dev. Int.* 22 1–36. 10.1108/CDI-04-2016-0058

[B15] Cui L. X. Jiang L. M. (2016). Impact of authentic leadership on employees’ individual initiative—based on the mediating role of leader-member exchange. *Sci. Technol. Economy* 29 75–79.

[B16] Eisenberger R. Armeli S. Rexwinkel B. Lynch P. D. Rhoades L. (2001). Reciprocation of perceived organizational support. *J. Appl. Psychol.* 86 42–51. 10.1037/0021-9010.86.1.42 11302232

[B17] Eisenberger R. Rhoades S. L. Wen W. Q. (2020). Perceived organizational support: why caring about employees counts. *Annu. Rev. Organizat. Psychol. Organizat. Behav.* 7 101–124. 10.1146/annurev-orgpsych-012119-044917

[B18] Fay D. Frese M. (2001). The concept of personal initiative(PI):An overview of validity studies. *Hum. Perform.* 14 97–124. 10.1207/S15327043HUP1401_06

[B19] Ferris G. R. Perrew P. L. Douglas C. (2002). Social effectiveness in organizations: Construct validity and research directions. *J. Leadersh. Organizat. Stud.* 9 49–63. 10.1177/107179190200900104

[B20] Ferris G. R. Treadway D. C. Kolodinsky R. W. Hochwarter W. A. Kacmar C. J. Douglas C. (2005). Development and validation of the political skill inventory. *J. Manag.* 31 126–152. 10.1177/0149206304271386

[B21] Ferris G. R. Treadway D. C. Perrewé P. L. Brouer R. L. Douglas C. Lux S. (2007). Political skill in organizations. *J. Manage.* 33 290–320. 10.1177/0149206307300813

[B22] Frese M. Fay D. Hilburger T. Leng K. Tag A. (1997). The concept of personal initiative: Operationlization, reliability and validity in two German samples. *J. Occupat. Organizat. Psychol.* 70 139–161. 10.1111/j.2044-8325.1997.tb00639.x

[B23] Fu R. P. (2020). Summary of the “14th Five-Year Plan” seminar on emergency planning-to promote the modernization of emergency management system and capacity under the guidance of emergency planning. *China Emerg. Manage.* 2020 14–19.

[B24] Gao J. Ma H. Y. (2008). Political skill in organizations and the relevant studies. *Adv. Psychol. Sci.* 2008 598–605.

[B25] Gao Y. (2020). Construction of evaluation system of public policy implementation capacity: acase study of Chinese national policy supporting college students’ entrepreneurship. *J. Wuhan Bus. Univers.* 34 78–82.

[B26] Geng X. Y. Cheng G. Y. (2021). Factors of emergency management capability of coal mine enterprises based on SEM. *Coal Enginee.* 53 184–188.

[B27] Gong L. (2019). *Effect mechanism of employee pay value on peripheral performance.* Ph. D. thesis. Lanzhou: Lanzhou University.

[B28] Greenglass E. R. Fiksenbaum L. (2009). Proactive coping, positive affect, and well-testing for mediation using path analysis. *Eur. Psychol.* 14 29–39. 10.1027/1016-9040.14.1.29

[B29] Ismail Z. Samad D. Zakaria H. (2012). Factorsinfluencing the implementation of a safety management system for construction sites. *Safety Sci.* 50 418–423. 10.1016/j.ssci.2011.10.001

[B30] Kong H. Guo F. Chen X. Chen D. W. (2020). A study on behavioral safety management of subway construction personnel based on “2-4” model. *Construct. Safety* 35 44–47.

[B31] Kurtessis J. N. Eisenberger R. Ford M. T. Buffardi L. C. Adis C. S. (2017). Perceived Organizational Support: A Meta-Analytic Evaluation of Organizational Support Theory. *J. Manage.* 43 1854–1884. 10.1177/0149206315575554

[B32] Lisbona A. Palaci F. Salanova M. Frese M. (2018). Theeffects of work engagement and self-efficacy on personal initiative and performance. *Psicothema* 30 89–96. 10.7334/psicothema2016.245 29363476

[B33] Liu C. H. Zhao L. Zhou B. Li Y. B. (2020). Analysis on the influencing factors of power safety management execution. *Popular Standardizat.* 2020 197–198.

[B34] Liu H. Q. Wei L. H. Wang F. J. Tang S. S. (2018). Growth need strength of supervisors & subordinates, incentive structure and employee creative outcomes. *Manage. World* 34 95–108.

[B35] Liu W. (2008). *The study on the present situation of work initiative, the antecedents and the cultivating measures of employees in enterprises.* Ph. D. thesis. Chongqing: Chongqing University.

[B36] Liu Z. Q. Deng C. J. Liao J. Q. Long L. R. (2015). Organizational support, perceived status and employees’ innovative behavior:perspective of employment diversity. *J. Manage. Sci. China* 18 80–94.

[B37] Lu N. Zhang X. (2016). Research status and development tendency of emergency management in domestic collieries. *Mining Process. Equip.* 44 6–9.

[B38] Ma J. Z. (2020). Analysis on the construction of China’s emergency management system from the perspective of fighting COVID-19. *J. Party School Nanjing Municipal Committ. CPC* 2020 68–71+98.

[B39] Ni W. F. Le R. Wan R. (2020). Research on countermeasures for financial institutions to enhance emergency management ability under crisis impact-based on analysis of “COVID-19” epidemic impact. *FinancialInstitut. Technol. Market* 27 140–143.

[B40] Qi H. Li Z. Q. Chen H. (2020). Influence of implementation characteristics on the effectiveness of coal mine safety management system. *Coal Enginee.* 52 192–196.

[B41] Qin H. (2016). The Research Progress of the government emergency management organizational coordination: a literature review. *Sci. Technol. Dev.* 388–393. 10.4324/9781315709420-31

[B42] Qiu L. Ma P. Y. Deng P. (2020). Thoughts on the emergency management of national treasury under the epidemic crisis. *FinTech Time* 28 98–100.

[B43] Rao W. F. Liang N. (2007). On implementary ability of emergency management. *J. Railway Enginee. Soc.* 110–114.

[B44] Ren X. R. Nie G. M. (2020). Analysis of the current situation and study on the countermeasures to improvethe emergency capacity of the middle-level administrative cadres in a Grade IIIgeneral hospital in the epidemic situation of COVID-19. *Chin. Hospit.* 24 42–43.

[B45] Rhoades L. Eisenberger R. (2002). Perceived organizational support: A review of the literature. *J. Appl. Psychol.* 87 698–714. 10.1037/0021-9010.87.4.698 12184574

[B46] Salisbury W. D. Chin W. W. Gopal A. Newsted P. R. (2002). Research report: better theory through measurement—developing a scale to capture consensus on appropriation. *Inform. Syst. Res.* 13 91–103. 10.1287/isre.13.1.91.93 19642375

[B47] Shu Q. (2020). *Error management climate and employees’adaptive performance : the role of psychological need satisfaction and personal initiative.* Ph. D. thesis. Hefei: Anhui University.

[B48] Sun S. van Emmerik H. I. (2015). Are proactive personalities always beneficial? Political skill as a moderator. *J. Appl. Psychol.* 100 966–975. 10.1037/a0037833 25180658

[B49] Sun X. L. Zhao S. M. (2019). Theimpact of CEO risk-taking propensity on corporate entrepreneurship: a moderated mediation model. *Sci. Sci. Manage. S. T.* 40 107–124.

[B50] Sun X. L. Zhao S. M. Bai X. M. (2018). Relationship among institutional support, top management team risk propensity and corporate entrepreneurship. *Sci. Res. Manage.* 39 123–130.

[B51] Tan J. K. Kong Y. (2020). Research on the relationship between psychological contract violation of newly-employed university students and job burnout. *J. China Univers. Labor Relat.* 34 67–78.

[B52] Teng J. A. (2014). “review of researches on personal initiative theory,” in *Proceedings of 2014 2nd International Conference on Psychology*, Vol. 50(GuangDong: Management and Social Science), 316–320.

[B53] Treadway D. C. Breland J. W. Williams L. M. Jeewon C. Jun Y. Ferris G. R. (2013). Social influence and interpersonal power in organizations: roles of performance and political skill in two studies. *J. Manage.* 39 1529–1553. 10.1177/0149206311410887

[B54] Wang T. (2012). *Study on evaluation and promotion strategy of safety management execution of coal enterprises in Hebei Province.* Master’s thesis. Hebei: Hebei University of engineering.

[B55] Wang H. W. (2018). The Establishment of Ministry of Emergency Management of the People’s Republic of China under the Concept of Modern Emergency Management:Significance, Challenges and Countermeasures. *Safety Secur.* 39 1–6.

[B56] Wang T. Y. (2015). *An empirical research ofevaluation on emergency management capability in petrochemical enterprises.* Ph. D. thesis. Harbin: Harbin Institute of technology.

[B57] Wang Y. Z. Zhao J. Y. (2020). The influence mechanism of perceived organizational support on employees’ constructive deviance. *J. Hubei Univers. Econom.* 18 74–81.

[B58] Wang Z. (2010). *Evaluation and improvement measures of safety management execution power on state-owned key minesin Shanxi - take M mine corporation for example.* Ph. D. thesis. Taiyuan: Taiyuan University of technology.

[B59] Wei L. Y. An D. D. (2020). The Influence of social support on job burnout-mediation of workplace mental power. *Manage. Observ.* 67–69.

[B60] Wei Z. J. Yin T. F. Wang L. J. (2019). Researchon the influence of safety integrity management on safety behavior - taking coal mine enterprises as an example. *J. Safety Sci. Technol.* 15 119–123.

[B61] Wu Q. Lei C. Q. (2019). A Review of Research on Emergency Plan Based on Bibliometrics and Co-word Analysis. *J. Henan Univers.* 59 35–43.

[B62] Xu Z. B. (2020). Analysis of problems and measures in chemical safety engineering. *Petrochem. Indust. Technol.* 27 214–215. 10.1155/2020/4923984

[B63] Yang W. S. Yang S. L. (2020). Distributed leadership, organizational support, and new generation employees’ proactive-reactive innovation behavior:based on the moderate of supervisor-subordinate guanxi and values fit. *J. Industr. Engine. Enginee. Manage.* 34 10–19.

[B64] Yang W. S. Mou J. Z. Li B. Wang J. Y. (2019). Relationship between proactive personality and employee behavior: the role of proactive socialization behavior and political skill. *J. Psychol. Sci.* 42 1448–1454.

[B65] Yang W. Yin X. (2019). Bibliometric analysis of research progress on emergency management in China (2009-2018) - and Discussion on the research status of emergency management in Tibet. *Theoret. Platform Tibetan Dev.* 61–65.

[B66] Yang Y. (2020). Therelationship between career resilience and employee’s active change behavior-intermediation of risk-taking tendency. *J. Taiyuan Urban Vocat. Coll.* 32–36.

[B67] Ye C. J. He B. Sun X. Liu B. (2020). The double-edged sword effect of inclusive leadership in the field of innovation:mediating of perceived acceptability of norm violation and moderation of proactive personality. *J. Technol. Econom.* 39 136–146.

[B68] Yu Q. J. (2019). Build a ternary drive model for efficient execution. *CHO Bus. Manage. Rev.* 76–83.

[B69] Yuan X. Y. (2012). *Study on promotion strategies of the execution of the production safety for DX coal mine.* Ph. D. thesis. Liaoning: Liaoning University of engineering and technology.

[B70] Zhang Z. G. Yu C. P. Li Y. J. (2016). The relationship among proactive personality, knowledge sharing and employee’s innovation behavior. *Manage. Rev.* 28 123–133.

[B71] Zhang A. Q. (2019). Problemsand countermeasures of lack of executive power in enterprise management. *China Circulat. Economy* 110–111.

[B72] Zhang X. Z. (2020). Application practice of safety management in coal mine mining engineering. *Petrochem. Industry Technol.* 27 196–197.

[B73] Zhang Y. P. Li J. Z. Feng G. R. Kang L. X. (2017). Study on mechanism of conflict management on emergency management level of mine. *Safety Coal Mines* 48 246–249.

[B74] Zhao K. Y. Qi J. Y. Zhao X. M. (2020). Research on influencing factors of teachers’ informationized teaching executive force. *e-Educat. Res.* 41 106–112.

[B75] Zhao Y. Yang X. Y. Wang J. H. Wang Y. X. (2020). Research on the relationships among knowledge sharing, proactive personality and new product development performance in state-owned enterprises. *Innovat. Technol.* 20 21–27.

[B76] Zhou C. (2018). *Researchon the influencing elements of execution of large sizedconstruction enterprises.* Ph. D. thesis. Xi’an: Xi’an University of science and technology.

